# COVID-19 Severity and Mortality Among Chronic Liver Disease Patients: A Systematic Review and Meta-Analysis

**DOI:** 10.5888/pcd19.210228

**Published:** 2022-08-25

**Authors:** Ramya Nagarajan, Yuvaraj Krishnamoorthy, Sathish Rajaa, Vishnu Shankar Hariharan

**Affiliations:** 1 Indian Council of Medical Research, National Institute of Epidemiology, Chennai, India; 2 Department of Community Medicine, ESIC Medical College and PGIMSR, K.K. Nagar, Chennai, Tamil Nadu, India; 3 Department of General Medicine, Hindu Mission Hospital, Chennai, Tamil Nadu, India

## Abstract

**Introduction:**

Pre-existing comorbid conditions in COVID-19 patients are risk factors for developing severe disease and death. We aimed to determine the association of chronic liver disease (CLD), a comorbid condition, with severity of disease and death among COVID-19 patients.

**Methods:**

We searched for studies reporting COVID-19 outcomes among CLD and non-CLD patients in databases including Medline, EMBASE, ScienceDirect, Google Scholar, and Cochrane Library from inception of the pandemic until February 2022. Risk of bias assessment was conducted by using the Newcastle-Ottawa Scale for assessing the quality of nonrandomized studies in meta-analyses. We conducted a meta-analysis with a random-effects model and reported pooled odds ratios (ORs) with 95% CIs.

**Results:**

We included 40 studies with 908,032 participants. Most studies were conducted in China and the US. COVID-19 patients with CLD had significantly higher odds of having a severe form of COVID-19 (pooled OR = 2.44; 95% CI, 1.89–3.16) and death (pooled OR = 2.35; 95% CI, 1.85–3.00) when compared with COVID-19 patients without CLD.

**Conclusion:**

The presence of CLD is significantly related to adverse clinical outcomes among COVID-19 patients in terms of severity and mortality. Clinicians should develop a comprehensive intervention plan to manage these high-risk patients and reduce COVID-19–related deaths.

**Summary**

**What is known on this topic?**

Pre-existing comorbid conditions in COVID-19 patients are risk factors for developing severe disease and death.

**What is added by this report?**

Our literature review indicated that chronic liver disease (CLD) is associated with increased adverse clinical outcomes in terms of severity of disease and death among COVID-19 patients.

**What are the implications for public health practice?**

Results of our meta-analysis should encourage clinicians worldwide to provide extra attention and intensive care for patients with underlying CLD who develop COVID-19.

## Introduction

A coronavirus is a group of viruses that causes mild to severe respiratory tract infections in humans and animals (1). In recent times, we have witnessed outbreaks of severe acute respiratory syndrome (SARS) virus (2004), Middle East Respiratory Syndrome coronavirus (MERS-CoV) (2012), and severe acute respiratory syndrome coronavirus 2 (SARS-CoV-2) (2019) that belong to this group of viruses (2). The SARS-CoV-2 outbreak is the most recent and was declared a global pandemic by the World Health Organization (WHO) on March 11, 2020 (3). As of May 16, 2021, 162 million cases and 3 million deaths were reported globally due to COVID-19 (4). The clinical features range from asymptomatic infection to severe pneumonia and death. However, patients who have comorbidities are more likely to have a severe form of the condition or to die (5).

Chronic liver disease (CLD) is marked by the gradual destruction of liver parenchyma over time. Various factors cause it; the most common are alcoholic liver disease, nonalcoholic fatty liver disease (NAFLD), chronic viral hepatitis, and genetic and autoimmune causes (6). Understanding the conditions that lead to severe disease and death among COVID-19–infected people is critical with the evolving pandemic (7). COVID-19 infection highlights the pre-existing weaknesses of the individual organ systems (8), making it logical to postulate that people with CLD may be susceptible to more severe respiratory infections or be at increased risk of death. In addition, it has been proposed that metabolic-associated fatty liver disease (MAFLD) or NAFLD is associated with significant or advanced fibrosis that might exacerbate the “cytokine storm” induced by the COVID-19 infection (9). The mechanism behind this is probably through the release of various proinflammatory hepatokines, which might contribute mechanistically to developing a severe form of COVID-19 infection (9). Several studies have found that hospitalized COVID-19 patients with CLD had an acute rise in liver enzymes, which results in a severe condition requiring mechanical ventilation and even leading to death (10–12). Existing evidence on COVID-19 outcomes among CLD patients has reported mixed results, making it difficult to determine a prognosis for these patients (10–13). Hence, we conducted a systematic review and meta-analysis to find the association between CLD and the severity of and mortality caused by COVID-19.

## Methods

This was a systematic review and meta-analysis of observational studies and was performed according to the Preferred Items for Systematic Reviews and Meta-Analyses (PRISMA) statement guidelines (14). The study protocol was registered in the PROSPERO database (registration ID: CRD42021291761).

### Eligibility criteria

We included studies with any of the following study designs: prospective or retrospective cohort, case control, and cross-sectional. Only published full-text studies were included; conference abstracts, unpublished data, and gray literature were excluded. Studies conducted among COVID-19 patients were included; studies among COVID-19 patients with comorbidities other than CLD were excluded.

Studies reporting the COVID-19 outcomes among CLD and non-CLD patients were included. CLD patients are diagnosed with the condition by clinical examination, laboratory or radiologic examination, or all 3 investigations. The CLD conditions most commonly found in COVID-19 patients included in our review were cirrhosis, viral hepatitis, NAFLD, and MAFLD. Studies reporting the diagnosis of CLD based on previous medical records were also included in the review.

Outcomes were the 1) severity of COVID-19 and 2) mortality due to COVID-19. The severity of the COVID-19 condition can be graded based on any of the following patient criteria: respiratory rate >30 breaths/min; oxygen saturation (SpO_2_) <93%; oxygenation index (PaO_2_/FiO_2_) ≤300 mm Hg; intensive care unit stay required; or mechanical ventilation (15). Studies reporting any of the outcomes mentioned above were included in our review.

### Search strategy

We conducted a comprehensive, systematic, and extensive search in the electronic databases Medline, EMBASE, ScienceDirect, Google Scholar, and Cochrane Library. We selected the terms required for the search during the protocol stage. We used both the medical subject headings (MeSH) and free-text words while searching these databases. The keywords and their synonyms were searched using appropriate truncations, wildcards, and proximity searching. The terms used to search were “liver disease”/exp OR “hepatic disease”:ti,ab OR “hepatic disorder”:ti,ab OR “hepatopathy”:ti,ab OR “liver cell disease”:ti,ab OR “liver disease”:ti,ab OR “liver diseases”:ti,ab OR “liver disorder”:ti,ab OR “liver illness”:ti,ab) AND “coronavirus disease 2019”/exp AND (“mortality”/exp OR “excess mortality” OR “mortality” OR “mortality model” OR “disease severity”/exp OR “disease severity” OR “illness severity” OR “severity, illness” OR “cause of death”/exp OR “cause of death” OR “cause, death” OR “death cause” OR “death caused” OR “mortality cause” OR “death”/exp OR “death” OR “mortality”. We also searched for crucial concepts using corresponding subject headings in each database. The last search was carried out by combining the individual search results using appropriate Boolean operators (“OR” and “AND”). The search was narrowed down using the available filters on the type of studies. We restricted the search from the inception of the pandemic to February 2022 and published in English only (Supplementary Table 1 available at: https://drive.google.com/drive/folders/1mVlexUbFzmHcfvi44LTFi18OmTnMZXtT?usp=sharing). Bibliographies of the retrieved articles were also hand-searched to identify any themes missed during the database search.

### Study selection process

This process involved 3 stages:

1. *Primary screening:* Two independent investigators (R.N. and Y.K.) performed preliminary screening of title, abstract, and keywords by executing the literature search. Full-text articles were retrieved for the studies shortlisted on the basis of the eligibility criteria.
2. *Secondary screening:* The same 2 investigators (R.N. and Y.K.) screened the full text of these retrieved studies and assessed them against the review’s eligibility criteria. Studies that satisfied all the eligibility criteria concerning design, participants, exposure, and outcome were included.
3. *Finalizing the study selection:* Disagreements during the screening process between the investigators were resolved. A final consensus on the inclusion of studies was reached with the help of another investigator (S.R.).

### Data extraction

Data were extracted manually from the included studies using a structured data extraction form that was developed and pilot tested during the protocol stage. We extracted the following data: general information, such as author and year of publication; information related to methods, such as study design, setting, sample size, sampling strategy, study participants, inclusion and exclusion criteria, outcome assessment method, and quality-related information; and information related to outcomes, such as patients’ severity of disease and mortality rates. Data were entered by the investigator (S.R.), and the entry was double checked by the secondary investigator (V.H.).

### Risk of bias assessment

Two independent investigators (S.R. and V.H.) used the Newcastle-Ottawa Scale to assess the risk of bias and quality of nonrandomized studies in meta-analyses under 3 domains: selection, comparability, and outcome (16). The quality of the study was graded as good, fair, or poor based on the scores obtained under each domain.

### Data synthesis

We used Stata version 16 (StataCorp LLC) to conduct the meta-analysis. Because all outcomes were dichotomous, the number of events and participants in each group were entered to obtain the pooled effect estimate in terms of odds ratios (ORs) with 95% CIs and prediction intervals (PIs). We used the random-effects model with the restricted maximum likelihood method to calculate the weights of individual studies (17) because of the clinical and methodologic heterogeneity among the included studies. We used the command *meta esize* to compute the summary statistic; it automatically adjusts for zero cells by adding 0.5 to all cells in a 2-by-2 table that contains a zero value while computing the summary statistic. Evidence of between-study variance due to heterogeneity was assessed through the ꭓ^2^ test of heterogeneity and *I* ^2^ statistics to quantify the inconsistency. *I* ^2^ less than 25% is mild, 25% to 75% is moderate, and more than 75% is considered substantial heterogeneity (17). Study-specific and pooled estimates were graphically represented through a forest plot. We also performed a sensitivity analysis to assess the robustness of the results by removing the studies one at a time and checking for any significant variation in the results. We also performed subgroup analysis on the basis of each type of CLD.

We conducted univariable meta-regression with the study-level characteristics using the *metareg* package in Stata. Publication bias was assessed for each outcome using the funnel plot and Doi plot for visual interpretation and Egger test and Luis Furuya-Kanamori asymmetry index (LFK index) for statistical interpretation (18). Asymmetry of the funnel plot and Doi plot and *P* value less than .10 in the Egger test indicates the possibility of publication bias. On the basis of the LFK index value, the possibility of publication bias was classified as no asymmetry (value within ±1), minor asymmetry (value out of ±1 but within ±2), and major asymmetry (value more than ±2) (18).

## Results

**Figure 1:**
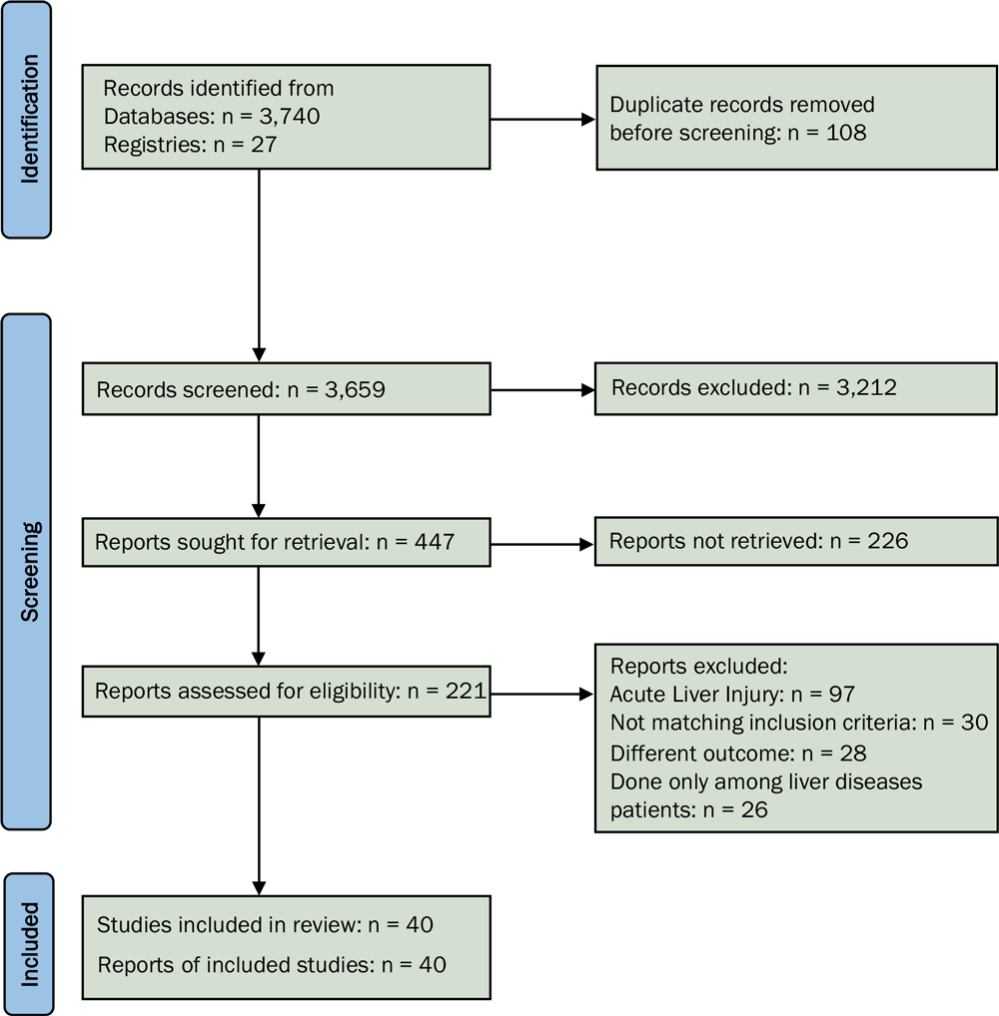
PRISMA flowchart showing the identification of studies for analysis of the association of chronic liver disease with severity of disease and mortality among COVID-19 patients. Abbreviation: PRISMA, Preferred Items for Systematic Reviews and Meta-Analyses.

We found 3,659 records through the systematic literature search and deemed 221 of those studies relevant for full-text retrieval. We also retrieved the full text for 36 articles obtained through manual searching of the bibliographies in the retrieved studies. During the second screening stage, 40 studies with 908,032 participants met the eligibility criteria and were included in the analysis (Figure 1) (8–12,15,19–52). This study was reported as per the PRISMA statement guidelines (Supplementary Table 2 available at: https://drive.google.com/drive/folders/1mVlexUbFzmHcfvi44LTFi18OmTnMZXtT?usp=sharing).

In total, 909,831 participants were found in the included studies, with a sample size ranging from 41 to 259,110 (Table). Among the 40 studies included, 15 reported on mortality due to COVID-19, 14 reported on the severity of COVID-19, and 11 reported both on severity and mortality. All included studies were retrospective; most studies were conducted in China (n = 14) and the US (n = 10). Half (21 of 40) of the included studies were low-quality (ie, per the Newcastle-Ottawa Scale) (Supplementary Table 3 available at: https://drive.google.com/drive/folders/1mVlexUbFzmHcfvi44LTFi18OmTnMZXtT?usp=sharing) (16).

**Table:** Characteristics of the Included Studies (N = 40)

Reference no.	Study	Country	Design	Mean age, y	Sample size	CLD criteria	COVID-19 severity criteria	Outcome assessed	Study quality
49	Afify et al (2021)	Egypt	RCS	NA	125	NA	ICU admission	Severity and mortality	Poor
9	Bahardoust et al (2021)	Iran	Case-control study	60	1,002	Previous medical records	Patients with respiratory rate >30 breaths/min, SpO_2_ <93% or PaO_2_/FiO_2_ ≤300 mm Hg	Severity	Good
10	Bajaj et al (2020)	US	Matched cohort study	61	145	Prior liver biopsy, evidence of frank hepatic decompensation, radiologic evidence of a nodular liver and/or features of portal hypertension or endoscopic evidence of varices	ICU transfer	Severity and mortality	Good
8	Berenguer et al (2020)	Spain	RCS	70	3,998	Previous medical records	NA	Mortality	Poor
12	Chen et al (2020)	China	RCS	62	274	Previous medical records	NA	Mortality	Poor
11	Frager et al (2021)	US	RCS	64.8	3,352	FIB-4 of >3.25 and/or Fibro Scan transient elastography results of >12.5 kPa	NA	Mortality	Poor
15	Galiero et al (2020)	Italy	RCS	65	618	Previous records and laboratory examination	NA	Mortality	Good
36	Gao et al (2020)	China	Cohort study	46	130	Presence of steatosis by histology or imaging	Patients with respiratory rate >30 breaths/min, SpO_2_ <93% or PaO_2_/FiO_2_ ≤300 mm Hg/mech ventilation/shock/ICU	Severity	Good
50	Ge et al (2021)	US	Cohort study	NA	38,387	Documentation of at least 1 OMOP concept identifier corresponding to previously validated ICD-10-CM codes for liver diseases at any time before the index date	NA	Severity and mortality	Good
43	Guan et al (2020)	China	RCS	47	1,099	Previous medical records	American Thoracic Society guidelines for community-acquired pneumonia	Severity	Poor
29	Guan et al (2020)	China	RCS	48.9	1,590	Previous medical records	American Thoracic Society guidelines for community-acquired pneumonia	Severity and mortality	Poor
35	Harrison et al (2020)	US	RCS	50	31,731	Previous medical records	NA	Mortality	Poor
51	Hashemi et al (2020)	US	RCS	63.4	363	Manual review of laboratory, imaging and/or histopathological data	ICU admission	Severity and mortality	Good
37	Huang et al (2020)	China	Cohort study	49	41	Laboratory investigation (LFT)	ICU admission	Severity	Poor
19	Ioannou et al (2020)	US	Cohort study	NA	10,131	Previous medical records	Need for mechanical ventilation	Severity and mortality	Good
23	Ji et al (2020)	China	Cohort study	44.5	202	Hepatic steatosis index (HSI = 8 × [ALT/ AST] + BMI [+2 if type 2 diabetes yes, +2 if female]) >36 points and/or by abdominal ultrasound examination	Patients with respiratory rate >30 breaths/min, SpO_2_ <93% or PaO_2_/FiO_2_ ≤300 mm Hg	Severity	Good
38	Kim et al (2020)	US	Cohort study	56.9	847	Previous medical records	NA	Mortality	Good
20	Lee et al (2020)	South Korea	Cohort study	61	1,005	Laboratory investigations	ICU admission	Severity and mortality	Good
40	Lei et al (2020)	China	Cohort study	56	5,771	Previous medical records	Patients with respiratory rate >30 breaths/min, SpO_2_ <93%	Severity	Fair
26	Li et al (2020)	China	Cohort study	59	104	Laboratory investigations	NA	Mortality	Poor
52	Mahamid et al (2020)	Israel	RCS	51	71	Radiologic examination	Patients with respiratory rate >30 breaths/min, SpO_2_ <93% or PaO_2_/FiO_2_ ≤300 mm Hg	Severity	Poor
24	Mallet et al (2021)	France	RCS	70	259,110	NA	Mechanical ventilation	Severity and mortality	Good
44	Mushtaq et al (2020)	Qatar	Case-control study	NA	589	HSI index of 36 and above	NA	Severity and mortality	Poor
39	Navarathnam et al (2021)	England	RCS	NA	91,541	Previous medical records	NA	Mortality	Good
45	Posso et al (2020)	Spain	RCS	78.2	834	Previous medical records	NA	Mortality	Fair
30	Rodriguez-Gonzalez et al (2021)	Spain	Case-control study	65	1,255	Laboratory investigations	NA	Mortality	Fair
41	Sarin et al (2020)	13 Asian countries	Cohort study	NA	228	Clinical and laboratory examination	Patients with respiratory rate >30 breaths/min, SpO_2_ <93% or PaO_2_/FiO_2_ ≤300 mm Hg	Severity and mortality	Poor
46	Schonfeld et al (2021)	Argentina	Cohort study	42.9	207,079	NA	ICU admission	Severity and mortality	Fair
27	Simon et al (2021)	Sweden	Cohort study	60.9	224,467	Liver biopsy	ICU admission	Severity	Good
47	Singh et al (2020)	US	Cohort study	NA	2,780	NA	NA	Mortality	Poor
31	Sun et al (2020)	China	Matched cohort study	47	63	Clinical and laboratory examination	Patients with respiratory rate >30 breaths/min, SpO_2_ <93% or PaO_2_/FiO_2_ ≤300 mm Hg; need for mechanical ventilation, ICU	Severity	Poor
42	Targher et al (2020)	China	Cohort study	NA	310	Laboratory investigations	ICU admission	Severity	Poor
21	de la Tijera et al (2021)	Mexico	Cross-sectional study	50.6	166	Previous medical records	Require invasive mechanical ventilation	Severity	Poor
33	Tobolowsky et al (2021)	US	Cohort study	83	101	NA	NA	Mortality	Poor
22	Veloz et al (2021)	Spain	Case- control study	NA	447	Historical medical records, radiology or analytic records within the last 24 months	NA	Mortality	Poor
25	Wang et al (2020)	China	Cohort study	69	339	Previous medical records	NA	Mortality	Good
48	Wang et al (2021)	US	RCS		16,960	Previous medical records	NA	Mortality	Poor
32	Yang et al (2020)	China	Cohort study	55	495	Laboratory investigations	Patients with respiratory rate >30 breaths/min, SpO_2_ <93% or PaO_2_/FiO_2_ ≤300 mm Hg	Severity	Poor
34	Zhang et al (2021)	China	Case- control study	47.9	172	Laboratory investigations	Patients with respiratory rate >30 breaths/min, SpO_2_ <93% or PaO_2_/FiO_2_ ≤300 mm Hg	Severity	Poor
28	Zhou et al (2020)	China	Cohort study	42.1	110	Previous medical records	COVID-19 management guidance 7th edition	Severity	Fair

Abbreviations: NA, not available; PaO_2_/FiO_2_, oxygenation index; RCS, retrospective cohort study; SpO_2_, oxygen saturation.

### Association between CLD and COVID-19 outcomes

#### Severity

**Figure 2:**
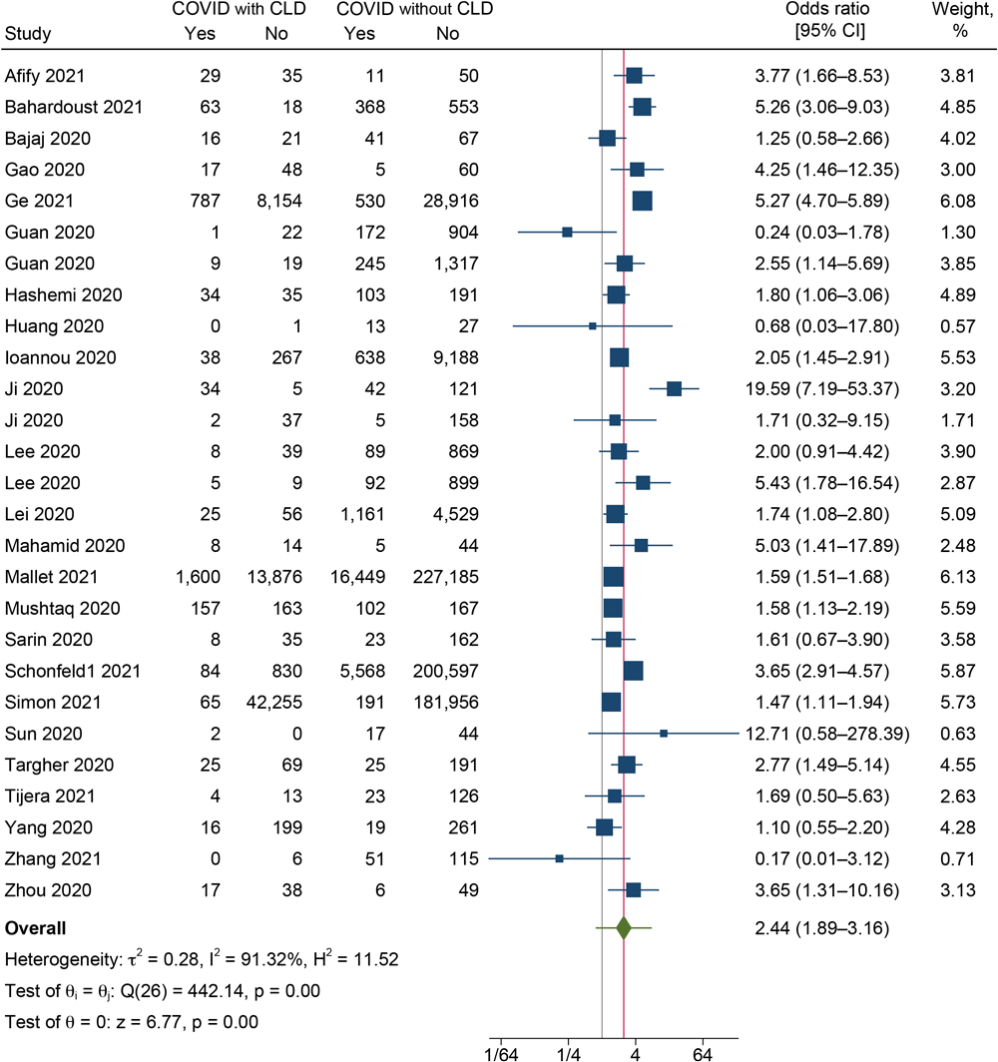
Forest plot showing the difference in severity between COVID-19 patients with and without CLD (N = 27). Abbreviation: CLD, chronic liver disease.

In our analysis, 25 studies reported the severity of the CLD and the non-CLD groups (9,10,19–21,23,24,27–29,31,32,34,36,37,40–44,46,49–52). The pooled OR was 2.44 (95% CI, 1.89–3.16; *I* ^2 =^ 91.3%; 95% PI, 0.79–7.55) (Figure 2), indicating that the odds of developing severe disease among COVID-19 patients with CLD were 2.44 times higher than among those without CLD. High heterogeneity was found between the studies reporting the severity outcome (*I* ^2^ = 91.3%, *P* < .001).

Subgroup analysis showed that COVID-19 patients with NAFLD had the highest odds of COVID-19 severity (pooled OR = 5.60; 95% CI, 1.52–20.64), followed by MAFLD (pooled OR = 3.20; 95% CI, 1.99–5.14) and cirrhosis (pooled OR = 3.09; 95% CI, 1.95–4.89) (Supplementary Figure 1 available at: https://drive.google.com/drive/folders/1mVlexUbFzmHcfvi44LTFi18OmTnMZXtT?usp=sharing). COVID-19 patients who had viral hepatitis did not have significantly higher odds of having a severe form of COVID-19 (pooled OR = 1.29; 95% CI, 0.36–4.63). Subgroup analysis by study design showed significantly higher odds of severity in the studies following cohort design (pooled OR = 3.10; 95% CI, 2.08–6.60; *P* = .001) (Supplementary Figure 2 available at: https://drive.google.com/drive/folders/1mVlexUbFzmHcfvi44LTFi18OmTnMZXtT?usp=sharing).

Results of the univariable meta-regression showed that geographic region, type of CLD, quality of study, year of publication, sample size, and mean age of participants were not significantly associated with the pooled effect size and cannot explain the substantial heterogeneity in the results (Supplementary Table 4 available at: https://drive.google.com/drive/folders/1mVlexUbFzmHcfvi44LTFi18OmTnMZXtT?usp=sharing).

Publication bias was graphically checked by funnel plot and Doi plot (Supplementary Figures 3 and 4 available at: https://drive.google.com/drive/folders/1mVlexUbFzmHcfvi44LTFi18OmTnMZXtT?usp=sharing). The funnel plot showed no sign of asymmetry, and it was also statistically proved by Egger test (*P* = *.*36); the Doi plot also showed no asymmetry, with an LFK index of 0.93. Sensitivity analysis showed no significant variation in the magnitude or direction of the outcome, indicating a lack of influence of a single study on the overall pooled estimate (Supplementary Figure 5 available at: https://drive.google.com/drive/folders/1mVlexUbFzmHcfvi44LTFi18OmTnMZXtT?usp=sharing).

#### Mortality

**Figure 3:**
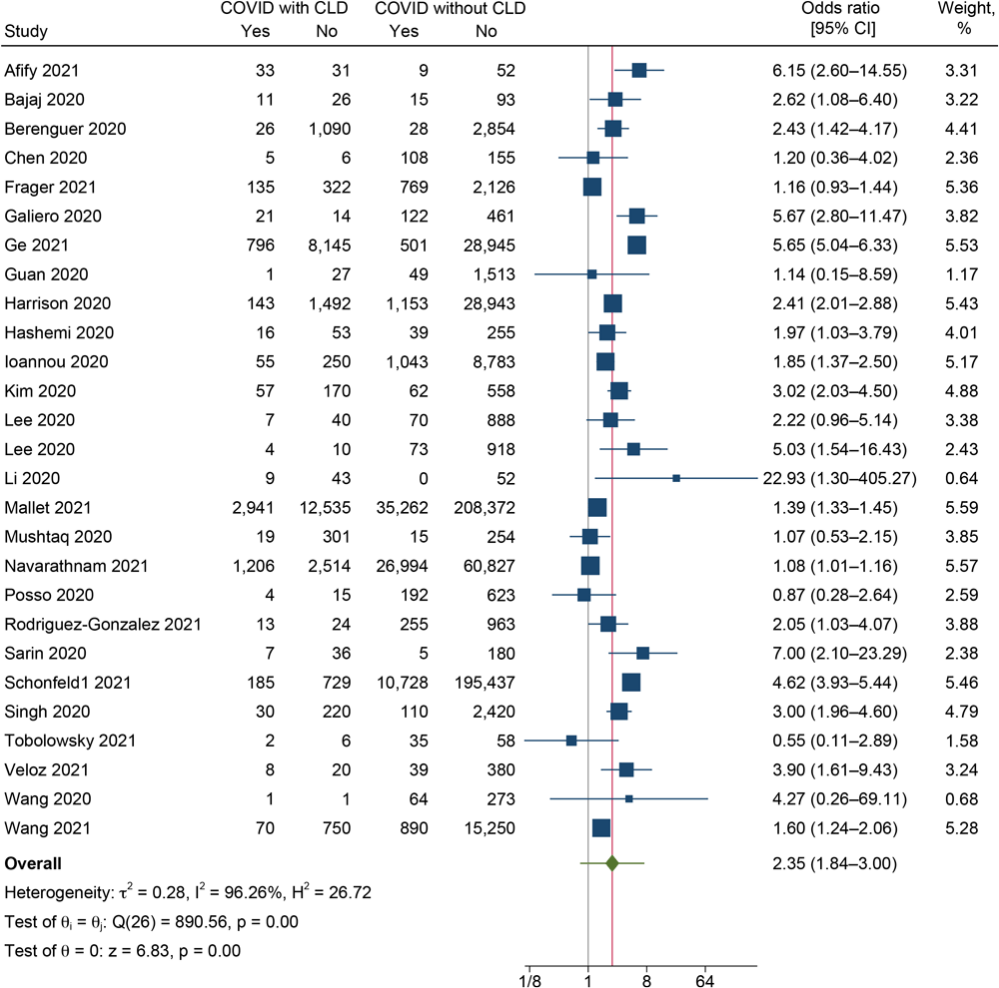
Forest plot showing the difference in mortality between COVID-19 patients with and without chronic liver disease (CLD) (N = 27).

In total, 26 studies reported on the mortality outcome among CLD and non-CLD patients (8,10–12,15,19,20,22,24–26,29,30,33,35,38,39,41,44–51). The pooled OR was 2.35 (95% CI, 1.84–3.00; *I* ^2^ = 96.26%; 95% PI, 0.76–7.18) (Figure 3), indicating that COVID-19 patients with CLD had 2.35 times higher odds of dying as patients without CLD. We found substantial heterogeneity between the studies reporting the mortality outcome (*I* ^2^ = 96.3%, *P* < *.*001).

Subgroup analysis based on the type of CLD could not be done because only cirrhosis had enough studies to give a pooled estimate (all the other studies reporting mortality outcomes were conducted among CLD patients without categorizing them based on the type of CLD). We found that COVID-19 patients with cirrhosis had 3.51 times higher odds of dying as patients without cirrhosis (pooled OR = 3.51; 95% CI, 2.41–5.10) (Supplementary Figure 6 available at: https://drive.google.com/drive/folders/1mVlexUbFzmHcfvi44LTFi18OmTnMZXtT?usp=sharing). Subgroup analysis by study design showed significantly higher odds of severity among the studies conducted using a cohort (pooled OR = 2.94; 95% CI, 2.09–4.13; *P* < .001) and a retrospective cohort design (pooled OR = 2.19; 95% CI, 1.51–3.17; *P* < .001) (Supplementary Figure 7 available at: https://drive.google.com/drive/folders/1mVlexUbFzmHcfvi44LTFi18OmTnMZXtT?usp=sharing).

Univariable meta-regression showed that only the mean age of the patients had a significant association with the pooled effect size (*P* = *.*01) and explained 48.3% of the between-study variability (Supplementary Figure 8 available at: https://drive.google.com/drive/folders/1mVlexUbFzmHcfvi44LTFi18OmTnMZXtT?usp=sharing). None of the other factors were significantly associated with the pooled effect size and cannot explain the substantial heterogeneity in the results (Supplementary Table 5 available at: https://drive.google.com/drive/folders/1mVlexUbFzmHcfvi44LTFi18OmTnMZXtT?usp=sharing).

Publication bias was graphically checked by funnel plot and Doi plot (Supplementary Figures 9 and 10 available at: https://drive.google.com/drive/folders/1mVlexUbFzmHcfvi44LTFi18OmTnMZXtT?usp=sharing). The funnel plot showed signs of asymmetry, with the Egger test (*P* = *.*10) also showing signs of possible publication bias. The Doi plot showed significant asymmetry, with an LFK index of 4.47. Sensitivity analysis showed no significant variation in the magnitude or direction of the outcome, indicating a lack of influence of a single study on the overall pooled estimate (Supplementary Figure 11 available at: https://drive.google.com/drive/folders/1mVlexUbFzmHcfvi44LTFi18OmTnMZXtT?usp=sharing).

## Discussion

We found that the risk of COVID-19 severity and death was twice as high among CLD patients than among non-CLD patients. Similar results were observed in a review conducted by Wu and Yang in which COVID-19 patients with CLD had more than 4 times the chance of developing severe disease and almost twice the chance of dying compared with non-CLD COVID-19 patients (53). Reviews conducted by Sharma et al and Yadav et al also found higher chances of developing severe disease and death among COVID-19 hospitalized patients with pre-existing liver diseases. Patients with elevated aspartate aminotransferase (AST) and alanine aminotransferase (ALT) were also reported to have higher chances of severe illness and death (54,55). However, a review conducted by Lippi et al states otherwise; no significant changes between liver disease and non–liver disease groups were found with respect to COVID-19 outcomes. However, the studies included in that analysis were limited, so its results should be interpreted cautiously (56).

Subgroup analysis based on the type of CLD showed that patients with NAFLD had the highest risk of severe disease, followed by those with MAFLD and cirrhosis. The estimates were also similar compared with the previous review findings (57–59). A similar analysis could not be done for mortality outcomes because of limitations in the number of studies. Still, research based on cirrhosis showed a higher effect size than the overall pooled estimate. Understanding the mechanism behind this finding is essential because it will help explain the reason for the association obtained in all the existing evidence. Subgroup analysis was also performed based on the study design adapted to conduct the study. We found higher odds of severity and mortality among studies adapting a cohort design. Though the estimates obtained from a cohort design are considered to be more powerful compared with a case-control or cross-sectional design, we are unclear about how the study design influences the severity and mortality outcome in our review (60). We recommend conducting further studies to evaluate the influence of study design in the outcome of severity and mortality studies.

The possible reason for the higher risk of severity among NAFLD patients could be the complex interplay of chronic active inflammatory pathways between the COVID-19–associated cytokine storm and NAFLD (59). Injury caused by the accumulation of fat in the liver could exacerbate the cytokine storm and worsen the prognosis of patients (61). In addition, liver fibrosis has been linked with a higher risk of severity among COVID-19 patients (62). Hence, liver fat accumulation and subsequent fibrosis may be the reasons for NAFLD patients’ more deficient outcomes. A similar mechanism was also observed for MAFLD because it was found to exacerbate the virus-induced inflammatory cytokine storm by increased reactive oxygen production and hepatic release of the proinflammatory cytokines in the COVID-19 patients (57,63). Finally, the possible pathogenesis behind the cirrhosis patients having a higher rate of severity and death following COVID-19 infection could be the excess systemic inflammation, intestinal dysbiosis, cirrhosis-induced immune dysfunction, and coagulopathies (59). Despite all these reasonings and mechanisms, determining the reason for such differential risk associated with different CLD patients is necessary. This determination can be achieved by performing proper longitudinal research in such patients and developing a deeper understanding of this issue.

The major strength of our review was the rigorous literature search and methodology followed to provide reliable estimates. In addition, this review adds to the limited evidence available on the prognostic importance of CLD among COVID-19 patients. We also performed additional subgroup analyses to stratify the risk of adverse outcomes based on the type of CLD and study design, meta-regression to explore the source of heterogeneity, and sensitivity analysis to check the robustness of our results.

Our study had limitations and findings should be interpreted cautiously, considering the difference in methods and quality across the included studies. Although the review by Mauvais-Jarvis et al stated the influence of gender over disease profile globally and the importance of having gender representation in medical research, our search found that data relevant to evaluating the severity of disease and mortality caused by COVID-19 in CLD patients by gender was lacking in the included studies, which is a limitation in our review (64). Our analysis also found significant between-study variability (significant χ^2^ test for heterogeneity and *I* ^2 ^statistics) for both outcomes. Such high heterogeneity can be attributed to the methodologic differences between the included studies, such as analysis by type of CLD, setting, sample size, and mean age. Meta-regression analysis did not indicate a significant source of heterogeneity for severity outcome and found only mean age as an essential source of heterogeneity for mortality outcome. In addition, we found substantial publication bias for the mortality outcome and found that most of the studies included in our review were of lower quality, which might further limit the generalizability of our study findings.

Although our results provide crucial information for better understanding the association of CLD and adverse COVID-19 outcomes, a need exists to perform longitudinal studies to establish the temporality of association and causal link between CLD and adverse clinical effects in the COVID-19 patients. Understanding this link will break a crucial barrier in managing COVID-19 patients and help prevent many deaths worldwide. 

Our findings have implications for clinical management. Although patients with any liver pathology have some adverse outcomes, the magnitude almost doubles if the patients have CLD. Results of our meta-analysis should encourage clinicians worldwide to provide extra attention and intensive care for patients with CLD, who should be provided with advanced management to prevent adverse clinical outcomes.
